# Supplementary material for: Psychosocial variables and quality of life during the COVID-19 lockdown: a correlational study on a convenience sample of young Italians
Source: PeerJ. 2020 Dec 18;8:e10611. doi: 10.7717/peerj.10611 (PMC7751426; doi:10.7717/peerj.10611)
Supplement: Supplemental Information 1 [file peerj-08-10611-s001.docx]

Covid-19

Start of Block: Introduzione

Gentile partecipante, i questionari che stai per compilare hanno l’obiettivo di comprendere come sia cambiata la qualità della vita durante la pandemia da Covid-19.  Lo studio si compone di alcune domande e di alcune scale che indagano la qualità della vita durante la situazione di emergenza che ci siamo trovati a fronteggiare. Il tempo complessivo è di circa 45 minuti. I questionari possono essere svolti anche in momenti diversi, l’importante è accedervi sempre dallo stesso dispositivo. Infatti, si può rispondere alle domande attraverso computer, tablet o cellulare (smartphone).   Prima di cominciare il questionario, ti verrà chiesto di lasciare la tua e-mail per essere ricontattato per partecipare ad una seconda fase della ricerca (quando finirà la pandemia). Ovviamente potrai decidere di non lasciare alcun contatto. In ogni caso, potrai, in futuro, scriverci per conoscere gli esisti della ricerca (laura.mandolesi@unina.it, andrea.chirico@uniroma1.it).   Le informazioni raccolte saranno utilizzate ai soli fini di ricerca e non avranno alcuna finalità clinico/diagnostica. Le informazioni verranno rese pubbliche solo su articoli scientifici come dati di gruppo, per cui il tuo contributo rimane anonimo. I dati saranno trattati con assoluta riservatezza secondo il regolamento (UE) 2016/679 GDPR (Regolamento (UE) 2016/679), Decreto legislativo n. 101/18. I Responsabili scientifici dello studio sono la prof.ssa Laura Mandolesi, Università di Napoli "Federico II", e il dr Andrea Chirico, Università di Roma "Sapienza".      È molto importante che tu risponda a TUTTE le domande, ricordando sempre che non esistono risposte giuste o risposte sbagliate, ma soltanto risposte che descrivono meglio il tuo modo di essere e le tue opinioni. Considera che le risposte che per prime vengono in mente, di solito, sono quelle che meglio ci descrivono. Per questo, ti consigliamo di rispondere in modo rapido a ciascuna domanda, senza pensarci troppo.    Ti ricordiamo che la partecipazione a questa ricerca è volontaria e che quindi puoi ritirarti o rinunciare in qualsiasi momento. Nella speranza che TU voglia collaborare, non ci resta che ringraziarti e augurarti

End of Block: Introduzione

Start of Block: Codice personale

| 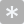 |
| --- |

È necessario che su ogni questionario sia presente un CODICE PERSONALE. Il Codice è indispensabile per le analisi statistiche in cui sono associati i questionari compilati in momenti differenti e deve essere identico nel caso in cui avvengano successive rilevazioni. Il codice garantisce a ciascun partecipante di rispondere sinceramente in modo anonimo e in nessun modo deve essere possibile risalire dal codice al nome della persona che ha risposto al questionario.  Al tempo stesso il codice deve essere facilmente ricordato (oltre che annotato) da ciascun partecipante. Per questo ti raccomandiamo di comporlo con 8 caratteri alfanumerici:   1) le prime 3 lettere del nome di Tua madre,  2) le prime 3 lettere del nome di Tuo padre,  3) il giorno del Tuo compleanno.
   Esempio:
 1) Nome della madre: FRA ncesca; 2) Nome del padre: GIO vanni
 3) Giorno del compleanno: 05 Aprile 
   Codice dell’esempio: FRAGIO05    Dunque ti preghiamo di inserire nell'apposito spazio il tuo codice personale seguendo le istruzioni appena esposte:

________________________________________________________________

Se vuoi continuare ad aiutarci in questo studio sulla qualità della vita durante la pandemia da Covid-19, ti chiediamo di inserire la tua e-mail. In questo modo potremo chiedere ancora la tua collaborazione quando questa emergenza sarà conclusa (FACOLTATIVO)

________________________________________________________________

End of Block: Codice personale

Start of Block: Socio-demografico (Socio-Dem)

Ti chiediamo di riempire gli appositi spazi o selezionare la risposta che reputi corretta

Età:

________________________________________________________________

Genere alla nascita:

- Maschio (1)
- Femmina (2)

Altezza (in cm):

________________________________________________________________

Peso:

________________________________________________________________

Studente:

- Sì (1)
- No (2)

Tipo di studente:

- Scuola superiore (1)
- Universitario (2)

Svolgi un'attività lavorativa?

- Sì (1)
- No (2)

Tipologia di attività lavorativa:

- Libero professionista (1)
- Impiegato (2)
- Attività lavorative saltuarie (p.e. cameriere, ripetizioni, babysitter) (3)
- Altro (4)

Regione in cui abiti durante la quarantena:

________________________________________________________________

Oltre a te, quante persone vivono nella tua abitazione durante la quarantena?

- 0 (9)
- 1 (1)
- 2 (2)
- 3 (3)
- 4 (4)
- 5 (5)
- Oltre 5 (6)

Hai un amico o un conoscente che è stato infettato dal Covid-19?

- Sì (5)
- No (6)

Indicare il numero di amici/conoscenti che hanno contratto il Covid-19

________________________________________________________________

Hai un familiare che è stato infettato dal Covid-19?

- Sì (5)
- No (6)

Indicare il numero di familiari che hanno contratto il Covid-19

________________________________________________________________

End of Block: Socio-demografico (Socio-Dem)

Start of Block: Stile di vita prima della quarantena (LBQ)

End of Block: Stile di vita durante la quarantena (LDQ)

Start of Block: Quality of Life (WHOQOL)

Le seguenti domande fanno riferimento a come ti senti in merito alla qualità della tua vita nelle ULTIME DUE SETTIMANE. Per qualità della vita si intende il tuo benessere emotivo, sociale e fisico, e la capacità di svolgere le tue attività (sociali, lavorative o sportive) durante la vita quotidiana.

| 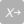 |
| --- |

Pensando alle ULTIME DUE SETTIMANE:

|  | Estremamente bassa (1) | Bassa (2) | Né bassa né alta (3) | Alta (4) | Estremamente alta (5) |
| --- | --- | --- | --- | --- | --- |
| Come giudicheresti la qualità della tua vita? (WHOQOL_1) |  |  |  |  |  |

| 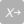 |
| --- |

Pensando alle ULTIME DUE SETTIMANE:

|  | Estremamente insoddisfatto (1) | Moderatamente insoddisfatto (2) | Né insoddisfatto né soddisfatto (3) | Moderatamente soddisfatto (4) | Estremamente soddisfatto (5) |
| --- | --- | --- | --- | --- | --- |
| Quanto sei soddisfatto della tua salute fisica? (WHOQOL_2) |  |  |  |  |  |

| 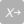 |
| --- |

Le seguenti domande si riferiscono a quanto hai provato determinate sensazioni nelle ULTIME DUE SETTIMANE:

|  | Per niente (1) | Poco (2) | Moderatamente (3) | Abbastanza (4) | Molto (5) |
| --- | --- | --- | --- | --- | --- |
| Fino a che punto senti che il dolore fisico ti impedisce di fare ciò che vuoi? (WHOQOL_3) |  |  |  |  |  |
| Quanto hai bisogno di un trattamento medico di qualsiasi tipo per svolgere normalmente la tua vita quotidiana? (WHOQOL_4) |  |  |  |  |  |
| Quanto ti piace goderti la vita? (WHOQOL_5) |  |  |  |  |  |
| In che misura ritieni che la tua vita abbia un significato? (WHOQOL_6) |  |  |  |  |  |
| Quanto riesci a concentrarti? (WHOQOL_7) |  |  |  |  |  |
| Quanto ti senti sicuro nella tua vita quotidiana? (WHOQOL_8) |  |  |  |  |  |
| Quanto ti senti sano a livello fisico? (WHOQOL_9) |  |  |  |  |  |

| 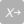 |
| --- |

Le seguenti domande si riferiscono a quanto sei stato in grado di fare o hai fatto determinate attività nelle ULTIME DUE SETTIMANE:

|  | Per niente (1) | Poco (2) | Moderatamente (3) | Abbastanza (4) | Molto (5) |
| --- | --- | --- | --- | --- | --- |
| Hai abbastanza energia per la vita di tutti i giorni? (WHOQOL_10) |  |  |  |  |  |
| Accettare il tuo aspetto fisico? (WHOQOL_11) |  |  |  |  |  |
| Hai abbastanza soldi per soddisfare le tue esigenze? (WHOQOL_12) |  |  |  |  |  |
| Quanto sono disponibili per te le informazioni di cui hai bisogno nella tua vita quotidiana? (WHOQOL_13) |  |  |  |  |  |
| In che misura hai l'opportunità di svolgere attività ricreative? (WHOQOL_14) |  |  |  |  |  |
| Quanto sei in grado di muoverti in maniera autonoma? (WHOQOL_15) |  |  |  |  |  |

| 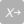 |
| --- |

Le seguenti domande ti chiedono di esprimere quanto, nelle ULTIME DUE SETTIMANE, ti sei sentito soddisfatto o insoddisfatto su alcuni aspetti della tua vita

|  | Estremamente insoddisfatto (1) | Moderatamente insoddisfatto (2) | Né insoddisfatto né soddisfatto (3) | Moderatamente soddisfatto (4) | Estremamente soddisfatto (5) |
| --- | --- | --- | --- | --- | --- |
| Quanto reputi soddisfacente il tuo sonno notturno? (WHOQOL_16) |  |  |  |  |  |
| Quanto sei soddisfatto della tua capacità di svolgere le tue attività quotidiane? (WHOQOL_17) |  |  |  |  |  |
| Quanto sei soddisfatto delle tue capacità lavorative? (WHOQOL_18) |  |  |  |  |  |
| Quanto sei soddisfatto di te stesso? (WHOQOL_19) |  |  |  |  |  |
| Quanto sei soddisfatto delle tue relazioni personali? (WHOQOL_20) |  |  |  |  |  |
| Quanto sei soddisfatto della tua vita sessuale? (WHOQOL_21) |  |  |  |  |  |
| Quanto sei soddisfatto del supporto che ti forniscono i tuoi amici? (WHOQOL_22) |  |  |  |  |  |
| Quanto sei soddisfatto del posto in cui vivi? (WHOQOL_23) |  |  |  |  |  |
| Quanto sei soddisfatto dell’accesso ai servizi sanitari? (WHOQOL_24) |  |  |  |  |  |
| Quanto sei soddisfatto dei mezzi di trasporto? (WHOQOL_25) |  |  |  |  |  |

| 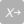 |
| --- |

La seguente domanda si riferisce alla frequenza con cui hai sentito o provato determinate sensazioni nelle ULTIME DUE SETTIMANE:

|  | Mai (1) | Raramente (2) | Occasionalmente (3) | Spesso (4) | Sempre (5) |
| --- | --- | --- | --- | --- | --- |
| Quanto spesso hai provato tristezza, ansia, disperazione o depressione? (WHOQOL_26) |  |  |  |  |  |

End of Block: Quality of Life (WHOQOL)

Start of Block: Covid Anxiety Items (CAI)

| 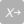 |
| --- |

Leggi attentamente le seguenti domande inerenti al Covid-19 e cerca di rispondere nella maniera più sincera possibile:

|  | Per niente (1) | Poco (2) | Moderatamente (3) | Abbastanza (4) | Molto (5) |
| --- | --- | --- | --- | --- | --- |
| Quanto sei preoccupato per il Covid-19? (CFI_1) |  |  |  |  |  |
| Quanto ritieni probabile che tu possa essere infettato dal Covid-19? (CFI_2) |  |  |  |  |  |
| Quanto è probabile che un tuo conoscente venga infettato dal Covid-19? (CFI_3) |  |  |  |  |  |
| Con quanta rapidità credi che si stia diffondendo il Covid-19? (CFI_4) |  |  |  |  |  |
| Quanto sei esposto alle informazioni inerenti al Covid-19? (CFI_5) |  |  |  |  |  |
| Qualora avessi contratto il Covid-19, quanto saresti preoccupato di essere gravemente malato? (CFI_6) |  |  |  |  |  |
| In che misura la minaccia del Covid-19 ha influenzato la tua decisione di stare con altre persone? (CFI_7) |  |  |  |  |  |
| In che misura la minaccia del Covid-19 ha influenzato i tuoi piani di viaggio? (CFI_8) |  |  |  |  |  |
| In che misura la minaccia del Covid-19 ha influenzato l'uso di comportamenti igienico-sanitari (es. lavarsi le mani)? (CFI_9) |  |  |  |  |  |

End of Block: Covid Anxiety Items (CAI)

| 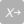 |
| --- |

Start of Block: Contamination Cognitions Scale (CCS-L-S)

Le seguenti domande prevedono una lista di oggetti sulla quale dovrai indicare in percentuale quanto ritieni probabile il contagio da Covid-19 **qualora venissi a contatto con essi (PROBABILITÀ) e la gravità di un eventuale contagio (GRAVITÀ),** indipendentemente dalla frequenza con cui vieni a contatto con tali oggetti:

|  | 0 | 10 | 20 | 30 | 40 | 50 | 60 | 70 | 80 | 90 | 100 |
| --- | --- | --- | --- | --- | --- | --- | --- | --- | --- | --- | --- |

| Maniglie toilette -PROBABILITÀ () | 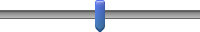 |
| --- | --- |
| Maniglie toilette - GRAVITÀ () | 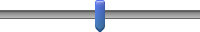 |
| Water - PROBABILITÀ () | 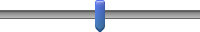 |
| Water - GRAVITÀ () | 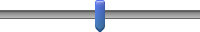 |
| Rubinetto lavandino - PROBABILITÀ () | 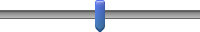 |
| Rubinetto lavandino - GRAVITÀ () | 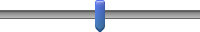 |
| Maniglie porte - PROBABILITÀ () | 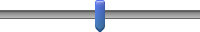 |
| Maniglie porte - GRAVITÀ () | 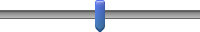 |
| Attrezzature allenamento - PROBABILITÀ () | 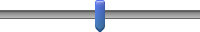 |
| Attrezzature allenamento - GRAVITÀ () | 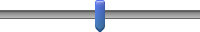 |
| Telefoni - PROBABILITÀ () | 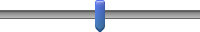 |
| Telefoni - GRAVITÀ () | 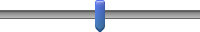 |
| Corrimano - PROBABILITÀ () | 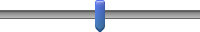 |
| Corrimano - GRAVITÀ () | 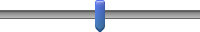 |
| Pulsanti ascensore - PROBABILITÀ () | 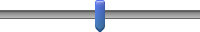 |
| Pulsanti ascensore - GRAVITÀ () | 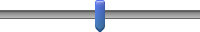 |
| Animali - PROBABILITÀ () | 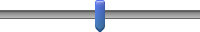 |
| Animali - GRAVITÀ () | 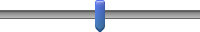 |
| Carne cruda - PROBABILITÀ () | 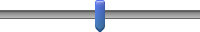 |
| Carne cruda - GRAVITÀ () | 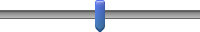 |
| Denaro - PROBABILITÀ () | 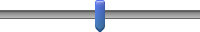 |
| Denaro - GRAVITÀ () | 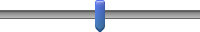 |
| Prodotti non lavati - PROBABILITÀ () | 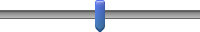 |
| Prodotti non lavati - GRAVITÀ () | 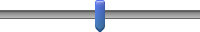 |
| Cibi toccati da altre persone - PROBABILITÀ () | 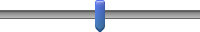 |
| Cibi toccati da altre persone - GRAVITÀ () | 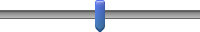 |

End of Block: Contamination Cognitions Scale (CCS-L-S)

Start of Block: Support for Public Health Initiatives to Reduce Spread of COVID-19 (RSC)

| 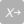 |
| --- |

Leggi attentamente le seguenti affermazioni e indica il tuo grado di accordo o disaccordo:

|  | Fortemente in disaccordo (1) | In disaccordo (2) | Leggermente in disaccordo (3) | Né in disaccordo né in accordo (4) | leggermente d'accordo (5) | D'accordo (6) | Fortemente d'accordo (7) |
| --- | --- | --- | --- | --- | --- | --- | --- |
| Le restrizioni sulla distanza sociale messe in atto per fermare la diffusione di Covid-19 stanno facendo più male che bene (RSC_1) |  |  |  |  |  |  |  |
| Dobbiamo dare la priorità al ritorno alla vita quotidiana il prima possibile, indipendentemente dalla diffusione di COVID-19 (RSC_2) |  |  |  |  |  |  |  |
| In questo momento la cosa più importante che possiamo fare è adottare tutte le misure possibili per fermare la diffusione di COVID-19 (RSC_3) |  |  |  |  |  |  |  |
| È essenziale che tutti pratichiamo rigorosamente il distanziamento sociale, fino a quando gli esperti sanitari non dichiarino diversamente (RSC_4) |  |  |  |  |  |  |  |

End of Block: Support for Public Health Initiatives to Reduce Spread of COVID-19 (RSC)

Start of Block: Social Identity Affirming Behaviors in Isolation (SIABI)

| 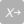 |
| --- |

Le seguenti domande fanno riferimento al periodo di quarantena che stiamo vivendo. **Indica quanto spesso hai messo in pratica i seguenti comportamenti DURANTE QUESTO PERIODO DI QUARANTENA:**

|  | Mai (1) | Raramente (2) | Occasionalmente (3) | Spesso (4) | Sempre (5) |
| --- | --- | --- | --- | --- | --- |
| Trovo nuovi modi creativi per mantenere la mia vecchia routine (ad es. Video chat con la famiglia e gli amici; lezioni di ginnastica online; attività culturali online) (SIABI_1) |  |  |  |  |  |
| Guardo o ascolto musica, video, film o riproduzioni di eventi culturali che per la maggior parte mi ricordano la cultura italiana (SIABI_2) |  |  |  |  |  |
| Condivido le cose con i miei amici e la mia famiglia al telefono o attraverso i social media che ci ricordano com'era la vita in Italia prima del COVID-19 (SIABI_3) |  |  |  |  |  |
| Faccio parte di gruppi utilizzando i social media o internet per sostituire quelli di cui non posso più farne parte di persona (SIABI_4) |  |  |  |  |  |
| Cerco di attuare comportamenti tipici della cultura italiana (ad es. cucino cibi che mi fanno sentire italiano) (SIABI_5) |  |  |  |  |  |

End of Block: Social Identity Affirming Behaviors in Isolation (SIABI)

Start of Block: Integrated COVID-19 Threat Scale (ICTS)

| 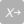 |
| --- |

Leggi attentamente la seguente lista e indica, secondo te, **quanto la pandemia da Covid-19 rappresenta una minaccia per:**

|  | Nessuna minaccia (1) | Poco minacciosa (2) | Moderatamente minacciosa (3) | Molto minacciosa (4) |
| --- | --- | --- | --- | --- |
| I diritti e la libertà del popolo italiano (ICTS_1) |  |  |  |  |
| Il significato di essere italiani (ICTS_2) |  |  |  |  |
| I valori e le tradizioni italiane (ICTS_3) |  |  |  |  |
| La democrazia italiana (ICTS_4) |  |  |  |  |
| Il mantenimento dell’ordine e delle leggi in Italia (ICTS_5) |  |  |  |  |
| La tua salute personale (ICTS_6) |  |  |  |  |
| La salute del popolo italiano nel suo insieme (ICTS_7) |  |  |  |  |
| L’economia italiana (ICTS_8) |  |  |  |  |
| La vita quotidiana nella tua comunità locale (ICTS_9) |  |  |  |  |

End of Block: Integrated COVID-19 Threat Scale (ICTS)

Start of Block: Social Connectedness (SCS) & Social Assicurance Scale (SAS)

Le seguenti domande fanno riferimento a come ti senti abitualmente. Ti chiediamo quindi di Indicare quanto sei d'accordo o in disaccordo con le seguenti affermazioni, considerando a come ti senti ABITUALMENTE, non in questo specifico periodo:

Mi sento disconnesso dal mondo che mi circonda

- Fortemente in disaccordo (1)
- In disaccordo (2)
- Leggermente in disaccordo (3)
- Leggermente d'accordo (4)
- D'accordo (5)
- Fortemente d'accordo (6)

Anche quando sono circondato da persone che conosco, non sento di farne parte

- Fortemente in disaccordo (1)
- In disaccordo (2)
- Leggermente in disaccordo (3)
- Leggermente d'accordo (4)
- D'accordo (5)
- Fortemente d'accordo (6)

Mi sento molto distante dalle persone

- Fortemente in disaccordo (1)
- In disaccordo (2)
- Leggermente in disaccordo (3)
- Leggermente d'accordo (4)
- D'accordo (5)
- Fortemente d'accordo (6)

Non sento alcuna vicinanza con i miei coetanei

- Fortemente in disaccordo (1)
- In disaccordo (2)
- Leggermente in disaccordo (3)
- Leggermente d'accordo (4)
- D'accordo (5)
- Fortemente d'accordo (6)

Non mi sento connesso con nessuno

- Fortemente in disaccordo (1)
- In disaccordo (2)
- Leggermente in disaccordo (3)
- Leggermente d'accordo (4)
- D'accordo (5)
- Fortemente d'accordo (6)

Mi capita di perdere la sensazione di essere connesso con la società

- Fortemente in disaccordo (1)
- In disaccordo (2)
- Leggermente in disaccordo (3)
- Leggermente d'accordo (4)
- D'accordo (5)
- Fortemente d'accordo (6)

Anche tra i miei amici non c’è un senso di fratellanza

- Fortemente in disaccordo (1)
- In disaccordo (2)
- Leggermente in disaccordo (3)
- Leggermente d'accordo (4)
- D'accordo (5)
- Fortemente d'accordo (6)

Non mi sento partecipe di alcun gruppo

- Fortemente in disaccordo (1)
- In disaccordo (2)
- Leggermente in disaccordo (3)
- Leggermente d'accordo (4)
- D'accordo (5)
- Fortemente d'accordo (6)

End of Block: Social Connectedness (SCS) & Social Assicurance Scale (SAS)

Start of Block: MOS Social Support Survey (MOS)

Nella seguente domanda, indica quanti amici intimi e parenti stretti hai (persone con cui ti senti a tuo agio e con cui puoi confidarti). Indicare il numero:

________________________________________________________________

Le persone a volte stanno con gli altri per compagnia, assistenza o altri tipi di supporto, se dovessi averne bisogno, **quanto spesso puoi fare affidamento su qualcuno in questo periodo di quarantena...**

|  | Mai (96) | Raramente (97) | Occasionalmente (98) | Spesso (99) | Sempre (100) |
| --- | --- | --- | --- | --- | --- |
| ...che ti aiuti se fossi “costretto a letto” (1084) |  |  |  |  |  |
| ...che ti ascolti quando hai bisogno di parlare (1085) |  |  |  |  |  |
| ...che ti dia buoni consigli se sei in crisi (1086) |  |  |  |  |  |
| ...che ti porti dal medico se ne hai bisogno (1087) |  |  |  |  |  |
| ...che ti mostri amore e affetto (1088) |  |  |  |  |  |
| ...con cui divertirti (1089) |  |  |  |  |  |
| ...che possa darti informazioni per aiutarti ad affrontare una determinata situazione (1090) |  |  |  |  |  |
| ...con cui confidarsi o parlare di te o dei tuoi problemi (1091) |  |  |  |  |  |
| ...che ti abbracci (1092) |  |  |  |  |  |
| ...con cui stare insieme per rilassarsi (1093) |  |  |  |  |  |
| ...che ti prepari un pasto se fossi impossibilitato a farlo (1094) |  |  |  |  |  |
| ...da cui desideri davvero un consiglio (1095) |  |  |  |  |  |
| ...che ti aiuti a distrarti e a non pensare (1096) |  |  |  |  |  |
| ...che ti aiuti nelle faccende quotidiane qualora fossi malato (1097) |  |  |  |  |  |
| ...con cui condividere le tue preoccupazioni e le tue paure (1098) |  |  |  |  |  |
| ...a cui rivolgersi per suggerimenti su come affrontare un problema personale (1099) |  |  |  |  |  |
| ...con cui fare qualcosa di divertente (1100) |  |  |  |  |  |
| ...che capisca i tuoi problemi (1101) |  |  |  |  |  |
| ...da amare e farti sentire desiderato (1102) |  |  |  |  |  |

End of Block: MOS Social Support Survey (MOS)

Start of Block: UCLA Loneliness Scale (UCLALS)

| 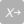 |
| --- |

Le frasi che seguono descrivono come le persone si sentono in alcuni momenti. Considerando il momento attuale, **per ciascuna frase indica quanto spesso:**

|  | Mai (1) | Raramente (2) | Spesso (4) | Sempre (5) |
| --- | --- | --- | --- | --- |
| Ti senti in sintonia con le altre persone (UCLALS_1) |  |  |  |  |
| Senti la mancanza di compagnia (UCLALS_2) |  |  |  |  |
| Senti che non hai nessuno a cui rivolgerti (UCLALS_3) |  |  |  |  |
| Ti senti solo (UCLALS_4) |  |  |  |  |
| Ti senti parte di un gruppo di amici (UCLALS_5) |  |  |  |  |
| Senti di avere qualcosa in comune con le persone che ti circondano (UCLALS_6) |  |  |  |  |
| Non ti senti vicino a qualcuno (UCLALS_7) |  |  |  |  |
| Senti che i tuoi interessi e le tue idee non vengono condivise dalle persone che ti circondano (UCLALS_8) |  |  |  |  |
| Ti senti estroverso ed amichevole (UCLALS_9) |  |  |  |  |
| Ti senti vicino alle persone (UCLALS_10) |  |  |  |  |
| Ti senti trascurato (UCLALS_11) |  |  |  |  |
| Ti senti che le relazioni con gli altri non abbiano significato (UCLALS_12) |  |  |  |  |
| Pensi che nessuno ti conosca veramente (UCLALS_13) |  |  |  |  |
| Ti senti isolato dagli altri (UCLALS_14) |  |  |  |  |
| Senti di poter trovare compagnia quando lo desideri (UCLALS_15) |  |  |  |  |
| Senti che ci sono persone che ti capiscono veramente (UCLALS_16) |  |  |  |  |
| Ti senti timido (UCLALS_17) |  |  |  |  |
| Percepisci che le persone sono intorno a te ma non sono con te (UCLALS_18) |  |  |  |  |
| Senti che ci sono persone con cui puoi parlare (UCLALS_19) |  |  |  |  |
| Senti di avere qualcuno a cui puoi rivolgerti (UCLALS_20) |  |  |  |  |

End of Block: UCLA Loneliness Scale (UCLALS)
