# Supplementary material for: Psychosocial variables and quality of life during the COVID-19 lockdown: a correlational study on a convenience sample of young Italians
Source: PeerJ. 2020 Dec 18;8:e10611. doi: 10.7717/peerj.10611 (PMC7751426; doi:10.7717/peerj.10611)
Supplement: Supplemental Information 2 [file peerj-08-10611-s002.docx]

Covid-19

Start of Block: Introduzione

Age:

________________________________________________________________

I am:

- Male(1)
- Female (2)

Height (in cm):

________________________________________________________________

Weight:

________________________________________________________________

Student:

- yes(1)
- no (2)

Do you attend:

- High school (1)
- University (2)

Do you have a job?

- yes(1)
- No (2)

Type of job:

- Freelance (1)
- Employee (2)
- Seasonal , or periodic job (waiter, babysitter etc..) (3)
- other(4)

Region where you lived during the panemic:

________________________________________________________________

How many people lived with you during the pandemic

- 0 (9)
- 1 (1)
- 2 (2)
- 3 (3)
- 4 (4)
- 5 (5)
- More than 5 (6)

Do you have a friend infected by COVID-19?

- Yes (5)
- No (6)

Number of friends infected

________________________________________________________________

Do you have a relative infected by COVID-19?

- Yes (5)
- No (6)

Number of relative infected

________________________________________________________________

The following questions refer to how you feel about the quality of your life in the LAST TWO WEEKS. Quality of life means your emotional, social and physical well-being, and the ability to carry out your activities (social, work or sports) during daily life.

| 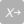 |
| --- |

Thinking about THE LAST TWO WEEKS:

|  | Very Poor (1) | Poor (2) | Neither poor nor good (3) | Good (4) | Very Good (5) |
| --- | --- | --- | --- | --- | --- |
| How would you rate your quality of life? (WHOQOL_1) |  |  |  |  |  |

| 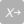 |
| --- |

Thinking about THE LAST TWO WEEKS:

|  | Very Dissatisfied  (1) | Dissatisfied (2) | Neither satisfied nor dissatisfied  (3) | Satisfied (4) | Very Satisfied (5) |
| --- | --- | --- | --- | --- | --- |
| How satisfied are you with your health?  (WHOQOL_2) |  |  |  |  |  |

| 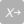 |
| --- |

The following questions ask about how much you have experienced certain things in THE LAST TWO WEEKS:

|  | Not at all  (1) | A  little  (2) | A moderate amount  (3) | Very much  (4) | An extreme amount  (5) |
| --- | --- | --- | --- | --- | --- |
| To what extent do you feel that physical pain prevents you from doing what you need to do? (WHOQOL_3) |  |  |  |  |  |
| How much do you need any medical treatment to function in your daily life? (WHOQOL_4) |  |  |  |  |  |
| How much do you enjoy life?  (WHOQOL_5) |  |  |  |  |  |
| To what extent do you feel your life to be meaningful? (WHOQOL_6) |  |  |  |  |  |
| How well are you able to concentrate?  (WHOQOL_7) |  |  |  |  |  |
| How safe do you feel in your daily life?  (WHOQOL_8) |  |  |  |  |  |
| How healthy is your physical environment?  (WHOQOL_9) |  |  |  |  |  |

| 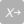 |
| --- |

The following questions ask about how completely you experience or were able to

do certain things in THE LAST TWO WEEKS:

|  | Not at all  (1) | A  little  (2) | Moderately  (3) | Mostly  (4) | Completely  (5) |
| --- | --- | --- | --- | --- | --- |
| Do you have enough energy for everyday life? (WHOQOL_10) |  |  |  |  |  |
| Are you able to accept your bodily appearance? (WHOQOL_11) |  |  |  |  |  |
| Have you enough money to meet your needs? (WHOQOL_12) |  |  |  |  |  |
| How available to you is the information that you need in your day-to-day life? (WHOQOL_13) |  |  |  |  |  |
| To what extent do you have the opportunity for leisure activities? (WHOQOL_14) |  |  |  |  |  |
| How well are you able to get around? (WHOQOL_15) |  |  |  |  |  |

| 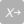 |
| --- |

The following questions ask you to say how **good or satisfied** you have felt about various aspects of your life over the LAST TWO WEEKS:

|  | Very Dissatisfied  (1) | Dissatisfied (2) | Neither satisfied nor dissatisfied  (3) | Satisfied (4) | Very Satisfied (5) |
| --- | --- | --- | --- | --- | --- |
| How satisfied are you with your sleep? (WHOQOL_16) |  |  |  |  |  |
| How satisfied are you with your ability to perform your daily living activities? (WHOQOL_17) |  |  |  |  |  |
| How satisfied are you with your capacity for work?  (WHOQOL_18) |  |  |  |  |  |
| How satisfied are you with yourself?  (WHOQOL_19) |  |  |  |  |  |
| How satisfied are you with your personal relationships? (WHOQOL_20) |  |  |  |  |  |
| How satisfied are you with your sex life? (WHOQOL_21) |  |  |  |  |  |
| How satisfied are you with the support you get from your friends? (WHOQOL_22) |  |  |  |  |  |
| How satisfied are you with the conditions of your living place?  (WHOQOL_23) |  |  |  |  |  |
| How satisfied are you with your access to health services? (WHOQOL_24) |  |  |  |  |  |
| How satisfied are you with your transport? (WHOQOL_25) |  |  |  |  |  |

| 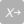 |
| --- |

The following question refers to how often you have felt or experienced certain things in THE LAST TWO WEEKS:

|  | Never  (1) | Seldom  (2) | Quite often  (3) | Very often  (4) | Always  (5) |
| --- | --- | --- | --- | --- | --- |
| How often do you have negative feelings such as blue mood, despair, anxiety,  depression? (WHOQOL_26) |  |  |  |  |  |

End of Block: Quality of Life (WHOQOL)

Start of Block: Covid Fear Inventory (CFI)

| 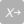 |
| --- |

Read carefully the following Covid-19 questions and try to answer them in the most sincere way possible:

|  | Very little  (1) | A  little  (2) | Moderately  (3) | Much  (4) | Very Much  (5) |
| --- | --- | --- | --- | --- | --- |
| To what extent are you concerned about Covid-19?  (CFI_1) |  |  |  |  |  |
| How likely is it that you could become infected with Covid-19?  (CFI_2) |  |  |  |  |  |
| How likely is it that someone you know could become infected with Covid-19?  (CFI_3) |  |  |  |  |  |
| How quickly do you believe contamination from Covid-19 is spreading?  (CFI_4) |  |  |  |  |  |
| How much exposure have you had to information about Covid-19?  (CFI_5) |  |  |  |  |  |
| If you did become infected with Covid-19, to what extent are you concerned that you will be severely ill?  (CFI_6) |  |  |  |  |  |
| To what extent has the threat of Covid-19influenced your decisions to be around people?  (CFI_7) |  |  |  |  |  |
| To what extent has the threat of Covid-19influenced your travel plans?  (CFI_8) |  |  |  |  |  |
| To what extent has the threat of Covid-19 influenced your use of safety behaviors (e.g., hand sanitizer)?  (CFI_9) |  |  |  |  |  |

End of Block: Covid Fear Inventory (CFI)

| 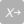 |
| --- |

Start of Block: Contamination Cognitions Scale (CCS-L-S)

The following questions provide a list of objects on which you have to indicate in percentage how likely you think the infection from Covid-19 if you come into contact with them (LIKELYHOOD) and the severity of a possible Infection (SEVERITY), regardless of how often you come into contact with such objects

|  | 0 | 10 | 20 | 30 | 40 | 50 | 60 | 70 | 80 | 90 | 100 |
| --- | --- | --- | --- | --- | --- | --- | --- | --- | --- | --- | --- |

| Toilet Handles - LIKELYHOOD () | 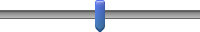 |
| --- | --- |
| Toilet Handles - SEVERITY () | 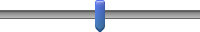 |
| Toilet Seats - LIKELYHOOD () | 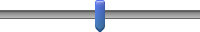 |
| Toilet Seats - SEVERITY () | 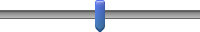 |
| Sink Faucets - LIKELYHOOD () | 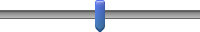 |
| Sink Faucets - SEVERITY () | 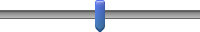 |
| Door Handles - LIKELYHOOD () | 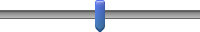 |
| Door Handles - SEVERITY () | 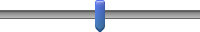 |
| Workout Equipment- LIKELYHOOD () | 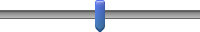 |
| Workout Equipment - SEVERITY () | 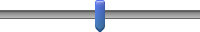 |
| Telephone Receivers - LIKELYHOOD () | 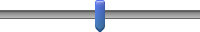 |
| Telephone Receivers - SEVERITY () | 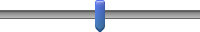 |
| Stairway Railings - LIKELYHOOD () | 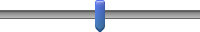 |
| Stairway Railings - SEVERITY () | 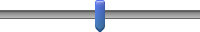 |
| Elevator Buttons - LIKELYHOOD () | 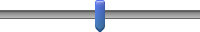 |
| Elevator Buttons - SEVERITY () | 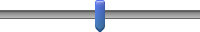 |
| Animals - LIKELYHOOD () | 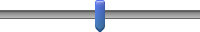 |
| Animals - SEVERITY () | 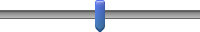 |
| Raw Meat - LIKELYHOOD () | 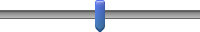 |
| Raw Meat - SEVERITY () | 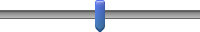 |
| Money - LIKELYHOOD () | 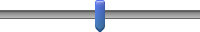 |
| Money - SEVERITY () | 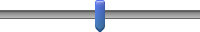 |
| Unwashed Produce - LIKELYHOOD () | 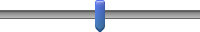 |
| Unwashed Produce - SEVERITY () | 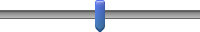 |
| Foods that others have touched - LIKELYHOOD () | 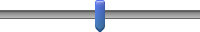 |
| Foods that others have touched - SEVERITY () | 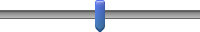 |

End of Block: Contamination Cognitions Scale (CCS-L-S)

Start of Block: Support for Public Health Initiatives to Reduce Spread of COVID-19 (RSC)

| 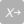 |
| --- |

Read carefully the following statements and indicate to what extent you agree or disagree

|  | Strongly Disagree  (1) | Disagree  (2) | Somewhat Disagree  (3) | Neither agree nor disagree (4) | Somewhat Agree (5) | Agree (6) | Strongly Agree  (7) |
| --- | --- | --- | --- | --- | --- | --- | --- |
| The social distancing restrictions being put into place to stop the spread of Covid-19 are doing more harm than good.  (RSC_1) |  |  |  |  |  |  |  |
| We need to prioritize going back to our normal routines as soon as possible, regardless of COVID-19’s spread.  (RSC_2) |  |  |  |  |  |  |  |
| Right now the most important thing we can do is take all measures possible to stop the spread of COVID-19.  (RSC_3) |  |  |  |  |  |  |  |
| It is essential that we strictly practice social distancing as a nation, until health care experts say otherwise.  (RSC_4) |  |  |  |  |  |  |  |

End of Block: Support for Public Health Initiatives to Reduce Spread of COVID-19 (RSC)

Start of Block: Social Identity Affirming Behaviors in Isolation (SIABI)

| 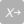 |
| --- |

The following questions refer to the quarantine period we are experiencing. Indicates how often you have done the following behaviours DURING THIS QUARANTINE PERIOD**:**

|  | Not at all (1) | Rarely  (2) | Sometimes  (3) | Often  (4) | Always  (5) |
| --- | --- | --- | --- | --- | --- |
| I find creative new ways to maintain my old routines (e.g., video chats with family and friends; online exercise classes; cultural activities online). (SIABI_1) |  |  |  |  |  |
| I watch or listen to music, videos, movies, or re-plays of cultural events that remind me most of Italian culture. (SIABI_2) |  |  |  |  |  |
| I share things with my friends and family on the phone or through social media that remind us of what life was like in Italy before COVID-19. (SIABI_3) |  |  |  |  |  |
| I engage with “virtual communities” through social media and online groups to replace the in-person communities I can no longer be a part of. (SIABI_4) |  |  |  |  |  |
| I engage in behaviors that I associate with Italian identity (e.g., I cook foods that make me feel Italian). (SIABI_5) |  |  |  |  |  |

End of Block: Social Identity Affirming Behaviors in Isolation (SIABI)

Start of Block: Integrated COVID-19 Threat Scale (ICTS)

| 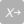 |
| --- |

Read carefully the following list and indicate, in your opinion, how much the pandemic from Covid-19 represents a threat to:

|  | Not a threat  (1) | Minor threat  (2) | Moderate threat (3) | Major threat  (4) |
| --- | --- | --- | --- | --- |
| The rights and freedoms of the Italian population as a whole (ICTS_1) |  |  |  |  |
| What it means to be Italian (ICTS_2) |  |  |  |  |
| Italian values and traditions (ICTS_3) |  |  |  |  |
| Italian democracy (ICTS_4) |  |  |  |  |
| The maintenance of law and order in Italy (ICTS_5) |  |  |  |  |
| Your personal health (ICTS_6) |  |  |  |  |
| The health of the Italian population as a whole (ICTS_7) |  |  |  |  |
| The Italian economy (ICTS_8) |  |  |  |  |
| Day-to-day life in your local community (ICTS_9) |  |  |  |  |

End of Block: Integrated COVID-19 Threat Scale (ICTS)

Start of Block: Social Connectedness (SCS) & Social Assicurance Scale (SAS)

The following questions refer to how you usually feel. We therefore ask you to indicate how much you agree or disagree with the following statements, considering how you USUALLY feel, not in this specific period:

I feel disconnected from the world around me

- Strongly disagree (1)
- Disagree (2)
- Somewhat disagree (3)
- Somewhat agree (4)
- Agree (5)
- Strongly agree (6)

Even around people I know, I don’t feel that I really belong

- Strongly disagree (1)
- Disagree (2)
- Somewhat disagree (3)
- Somewhat agree (4)
- Agree (5)
- Strongly agree (6)

I feel so distant from people

- Strongly disagree (1)
- Disagree (2)
- Somewhat disagree (3)
- Somewhat agree (4)
- Agree (5)
- Strongly agree (6)

I have no sense of togetherness with my peers

- Strongly disagree (1)
- Disagree (2)
- Somewhat disagree (3)
- Somewhat agree (4)
- Agree (5)
- Strongly agree (6)

I don’t feel related to anyone

- Strongly disagree (1)
- Disagree (2)
- Somewhat disagree (3)
- Somewhat agree (4)
- Agree (5)
- Strongly agree (6)

I catch myself losing all sense of connectedness with society

- Strongly disagree (1)
- Disagree (2)
- Somewhat disagree (3)
- Somewhat agree (4)
- Agree (5)
- Strongly agree (6)

Even among my friends, there is no sense of brother/sisterhood

- Strongly disagree (1)
- Disagree (2)
- Somewhat disagree (3)
- Somewhat agree (4)
- Agree (5)
- Strongly agree (6)

I don’t feel I participate with anyone or any group

- Strongly disagree (1)
- Disagree (2)
- Somewhat disagree (3)
- Somewhat agree (4)
- Agree (5)
- Strongly agree (6)

End of Block: Social Connectedness (SCS) & Social Assicurance Scale (SAS)

Start of Block: MOS Social Support Survey (MOS)

Next are some questions about the support that is available to you.

About how many close friends and close relatives do you have (people you feel at easewith and can talk to about what is on your mind)?

(write in number)

People sometimes look to others for companionship, assistance, or other types of support. How often is each of the following kinds of support available to you if you need it

|  | None of the time (96) | A little of the time (97) | Some of the time (98) | Most of the time (99) | All of the time (100) |
| --- | --- | --- | --- | --- | --- |
| Someone to help you if you were confined to bed  (1084) |  |  |  |  |  |
| Someone you can count on to listen to you when you need to talk  (1085) |  |  |  |  |  |
| Someone to give you good advice about a crisis  (1086) |  |  |  |  |  |
| Someone to take you to the doctor if you needed it  (1087) |  |  |  |  |  |
| Someone who shows you love and affection  (1088) |  |  |  |  |  |
| Someone to have a good time with  (1089) |  |  |  |  |  |
| Someone to give you information to help you understand a situation  (1090) |  |  |  |  |  |
| Someone to confide in or talk to about yourself or your problems  (1091) |  |  |  |  |  |
| Someone who hugs you  (1092) |  |  |  |  |  |
| Someone to get together with for relaxation  (1093) |  |  |  |  |  |
| Someone to prepare your meals if you were unable to  do it yourself  (1094) |  |  |  |  |  |
| Someone whose advice you really want  (1095) |  |  |  |  |  |
| Someone to do things with to help you get your mind  off things  (1096) |  |  |  |  |  |
| Someone to help with daily chores if you were sick  (1097) |  |  |  |  |  |
| Someone to share your most private worries and fears with  (1098) |  |  |  |  |  |
| Someone to turn to for suggestions about how to deal with a personal problem  (1099) |  |  |  |  |  |
| Someone to do something enjoyable with  (1100) |  |  |  |  |  |
| Someone who understands your problems  (1101) |  |  |  |  |  |
| Someone to love and make you feel wanted  (1102) |  |  |  |  |  |

End of Block: MOS Social Support Survey (MOS)

Start of Block: UCLA Loneliness Scale (UCLALS)

| 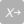 |
| --- |

The following statements describe how people sometimes feel. Considering the current moment, for each sentence it indicates:

|  | Never (1) | Rarely (2) | Sometimes (4) | Always (5) |
| --- | --- | --- | --- | --- |
| How often do you feel that you are “in tune” with the people around you? (UCLALS_1) |  |  |  |  |
| How often do you feel that you lack companionship? (UCLALS_2) |  |  |  |  |
| How often do you feel that there is no one you can turn to? (UCLALS_3) |  |  |  |  |
| How often do you feel alone? (UCLALS_4) |  |  |  |  |
| How often do you feel part of a group of friends? (UCLALS_5) |  |  |  |  |
| How often do you feel that you have a lot in common with the people around you? (UCLALS_6) |  |  |  |  |
| How often do you feel that you are no longer close to anyone? (UCLALS_7) |  |  |  |  |
| How often do you feel that your interests and ideas are not shared by those around you? (UCLALS_8) |  |  |  |  |
| How often do you feel outgoing and friendly? (UCLALS_9) |  |  |  |  |
| How often do you feel close to people? (UCLALS_10) |  |  |  |  |
| How often do you feel left out? (UCLALS_11) |  |  |  |  |
| How often do you feel that your relationship with others are not meainingful? (UCLALS_12) |  |  |  |  |
| How often do you feel that no one really knows you well? (UCLALS_13) |  |  |  |  |
| How often do you feel isolated from others? (UCLALS_14) |  |  |  |  |
| How often do you feel you can find companionship when you want? (UCLALS_15) |  |  |  |  |
| How often do you feel that there are people who really understand you? (UCLALS_16) |  |  |  |  |
| How often do you feel shy? (UCLALS_17) |  |  |  |  |
| How often do you feel that people are around you but not with you? (UCLALS_18) |  |  |  |  |
| How often do you feel that there are people you can talk to? (UCLALS_19) |  |  |  |  |
| How often do you feel that there are people you can turn to? (UCLALS_20) |  |  |  |  |

End of Block: UCLA Loneliness Scale (UCLALS)
